# The Relationship Between Beta-Blocker Use and Prognosis of Patients With Out-of-Hospital Cardiac Arrest

**DOI:** 10.7759/cureus.73949

**Published:** 2024-11-18

**Authors:** Kazunori Fukushima, Makoto Aoki, Nobuya Kitamura, Takashi Tagami, Hideo Yasunaga, Shotaro Aso, Yoshihisa Tateishi, Yusuke Sawada, Kiyohiro Oshima

**Affiliations:** 1 Department of Emergency Medicine, Gunma University Graduate School of Medicine, Maebashi, JPN; 2 Emergency Department, National Defense Medical College, Saitama, JPN; 3 Department of Emergency and Critical Care Medicine, Kimitsu Chuo Hospital, Kisarazu, JPN; 4 Department of Emergency and Critical Care Medicine, Nippon Medical School Musashi Kosugi Hospital, Kawasaki, JPN; 5 Department of Clinical Epidemiology and Health Economics, The University of Tokyo, Tokyo, JPN; 6 Department of Real-World Evidence, The University of Tokyo, Tokyo, JPN; 7 Department of Emergency and Critical Care Medicine, Chiba Kaihin Municipal Hospital, Chiba, JPN

**Keywords:** beta-blocker, japan, neurological outcomes, out-of-hospital cardiac arrest, prognosis, survival

## Abstract

Background: Out-of-hospital cardiac arrest (OHCA) has a poor prognosis. Patients with shockable rhythms often have better outcomes than those with nonshockable rhythms. A previous study reported a decline in shockable rhythms and poorer outcomes with the use of beta-blockers before OHCA. This study aimed to investigate the association between beta-blocker use and outcomes in OHCA patients using data from a multicenter prospective observational study in Japan.

Patients and methods: This study is a post hoc analysis based on data from the Survey of Survivors after Out-of-Hospital Cardiac Arrest in Kanto Area 2017 study, which included 9,909 OHCA patients in Japan. Patients aged 18 years or older with cardiogenic OHCA were included in the analysis, which involved multiple imputation and overlap weighting with propensity scores. As a subgroup analysis, data were extracted for patients with a history of cardiovascular disease and who were also subjected to multiple imputations and overlapping weighting. The outcomes were survival and favorable neurological outcomes at 30 days.

Results: Out of the 5,392 analyzed patients, 96 were taking beta-blockers before OHCA, and 5,296 were not. After adjusting for confounding factors using overlap weighting, beta-blocker use was not found to be associated with increased survival (odds ratio, OR, 1.07; 95% confidence interval, CI, 0.64-1.81) and favorable neurological outcomes (OR, 1.09; 95% CI, 0.61-1.95). The analysis of patients with a history of cardiovascular disease also showed no significant difference in survival based on beta-blocker use.

Conclusion: In this study, beta-blocker use was not associated with survival and favorable neurological outcomes in OHCA patients.

## Introduction

Out-of-hospital cardiac arrest (OHCA) is a severe medical condition with a poor prognosis [1]. The survival rate of OHCA is less than 10% and has remained relatively unchanged for the past 30 years [2]. To improve the prognosis of cardiac arrest, ongoing research studies have focused on favorable and unfavorable OHCA outcomes as well as more effective resuscitation methods.

The initial rhythm can serve as a prognostic marker, and an initial shockable rhythm, such as ventricular fibrillation (VF) or pulseless ventricular tachycardia, has a better prognosis than pulseless electrical activity and asystole [3]. In recent years, there has been a decreasing trend in shockable rhythms [4,5]. Certain studies have suggested a potential association between beta-blocker use and the reduced incidence of shockable rhythms during OHCA [6,7]. However, other investigations have provided conflicting results, with some reporting no significant association between beta-blocker use and initial rhythms [8]. Thus, it remains unclear whether beta-blockers can influence the initial rhythm. Furthermore, their impact on the prognosis of OHCA patients is not yet fully understood.

Beta-blockers are widely prescribed for patients with hypertension or arrhythmias and those recovering from myocardial infarction and heart failure since they can improve the long-term prognosis [9-13]. In Japan, which has become a super-aged society, the number of patients taking beta-blockers has increased. We hypothesized that beta-blocker use may influence survival or neurological outcomes in OHCA patients. Therefore, we examined the association between beta-blocker use and outcomes in OHCA patients using data from a multicenter prospective observational study in Japan.

## Materials and methods

Study design and setting

Our study involved a post hoc analysis, where we analyzed patients included in the Survey of Survivors after Out-of-Hospital Cardiac Arrest in Kanto Area (SOS-KANTO) 2017 study, a multicenter prospective observational registry of OHCA patients in the Kanto region of Japan. With the support of the Japanese Association for Acute Medicine of KANTO, the SOS-KANTO Study Group prospectively collected data on all OHCA patients who were transported by emergency medical services (EMS) to a participating facility in 2002. This study group conducted two prospective observational studies based on preregistered research themes [1,14]. The SOS-KANTO 2017 study included 9,909 cardiac arrest patients who received emergency treatment at 42 emergency medical centers between September 2019 and March 2021. We obtained approval from the institutional review board of Gunma University Hospital, Maebashi, Japan (HS2019-004), before participating in the SOS-KANTO 2017 study. Additionally, this study was conducted using anonymized data provided by the research office, ensuring no personally identifiable information was included. Informed consent was waived because of the anonymous nature of the data used.

Our study included OHCA patients aged ≧18 years who presented with a cardiogenic etiology. Beta-blocker use was determined based on medical records that were obtained and confirmed by EMS personnel during patient transport or upon hospital arrival and subsequently verified by hospital medical staff using the patient's medication history. In the SOS-KANTO study, the causes of cardiac arrest were classified as heart disease, internal causes (non-heart disease), and external causes. Internal causes refer to nontraumatic medical conditions that triggered the cardiac arrest. External causes refer to cardiac arrests resulting from trauma, accidents, drug overdoses, drowning, asphyxiation, or any other external injury or insult. For this study, we extracted patients whose cardiac arrest was believed to be caused by heart disease. The cause of cardiac arrest was recorded by the attending physicians at each participating facility. The exclusion criteria were as follows: no attempts at resuscitation and missing data about beta-blockers or outcomes.

Data collection and definitions

Data collection for the SOS-KANTO 2017 study has been reported previously [15]. EMS personnel collected prehospital patient information based on the Utstein format, and physicians in the participating facilities provided inhospital data, including inspection results, treatment, and prognosis. The attending physicians at each participating facility classified the cause of cardiac arrest as acute coronary syndrome, other heart disease, or presumed cardiogenic etiology, and collected information on patient survival at 30 days. These data were collected prospectively.

Our study used the following variables: age, sex, presence of witnesses, presence of bystander cardiopulmonary resuscitation (CPR), initial waveform, cause of cardiac arrest, Clinical Frailty Scale, medical history (presence of myocardial infarction, congestive heart failure, peripheral vascular disease, cerebrovascular disease, diabetes, hypertension, and atrial fibrillation), beta-blocker use, survival at 30 days, and the cerebral performance categories (CPCs) at 30 days. Preillness frailty in patients was evaluated using the Clinical Frailty Scale [16,17]. We divided the patients into those with and without beta-blocker use before OHCA. Information regarding beta-blocker use was obtained as part of the patient's medication history and was initially collected by EMS personnel during transport or by medical staff at each participating hospital facility through a review of medical records. In cases where the medication information was absent or ambiguous, these were recorded as missing data.

Outcome definitions

The primary outcome was survival at 30 days. The secondary outcomes were favorable neurological outcomes at 30 days. The neurological prognosis was evaluated using the CPC as follows: CPC 1, good performance; CPC 2, moderate disability; CPC 3, severe disability; CPC 4, comatose or persistent vegetative status; and CPC 5, brain death or death [18]. CPC 1 and CPC 2 were considered as favorable neurological outcomes.

Statistical analysis

The patients' baseline characteristics were examined using the t-test and chi-squared tests to compare the means (standard deviation, SD) and categorical variables, respectively. In cases where the continuous variables did not exhibit normality, the Mann-Whitney U test, a nonparametric alternative, was used to compare between groups. Additionally, all missing data without primary outcomes were imputed by multiple imputations using the assumption that data were missing at random [19]. We used multiple imputations to create and analyze 20 datasets. Each parameter was estimated in each imputed dataset separately and combined using Rubin's rules [19]. The covariates for multiple imputation and propensity score calculation were chosen using prior knowledge and possible predictors for outcomes in patients with OHCA [1,2,6-8]. We conducted overlap weighting, where more weight was assigned to individuals with a higher probability of belonging to both treatment groups, thereby enhancing the covariate balance between the beta-blocker and non-beta-blocker groups. The propensity score for overlap weighting was calculated using multivariate logistic regression analysis with the following variables: age, sex, witnessed arrest, bystander CPR, initial rhythm, Clinical Frailty Scale score, existing disease (myocardial infarction, congestive heart failure, peripheral vascular disease, cerebrovascular disease, diabetes, renal disease, hypertension, and atrial fibrillation), and CPC. As a sensitivity analysis, propensity score matching was also performed 1:1 using nearest neighbor matching of the propensity score.

Furthermore, as a subgroup analysis, we extracted the data of patients with a history of cardiovascular disease, such as myocardial infarction, congestive heart failure, hypertension, and atrial fibrillation. We conducted a similar analysis that involved multiple imputation and overlap weighting.

 All p values were two-sided, and a p value of 0.05 or less was considered statistically significant. All statistical analyses were performed with R (version 4.2.0; R Foundation for Statistical Computing, Vienna, Austria). The default settings of the mice 3.15.0 package were used for the multiple imputations.

## Results

**Figure 1:**
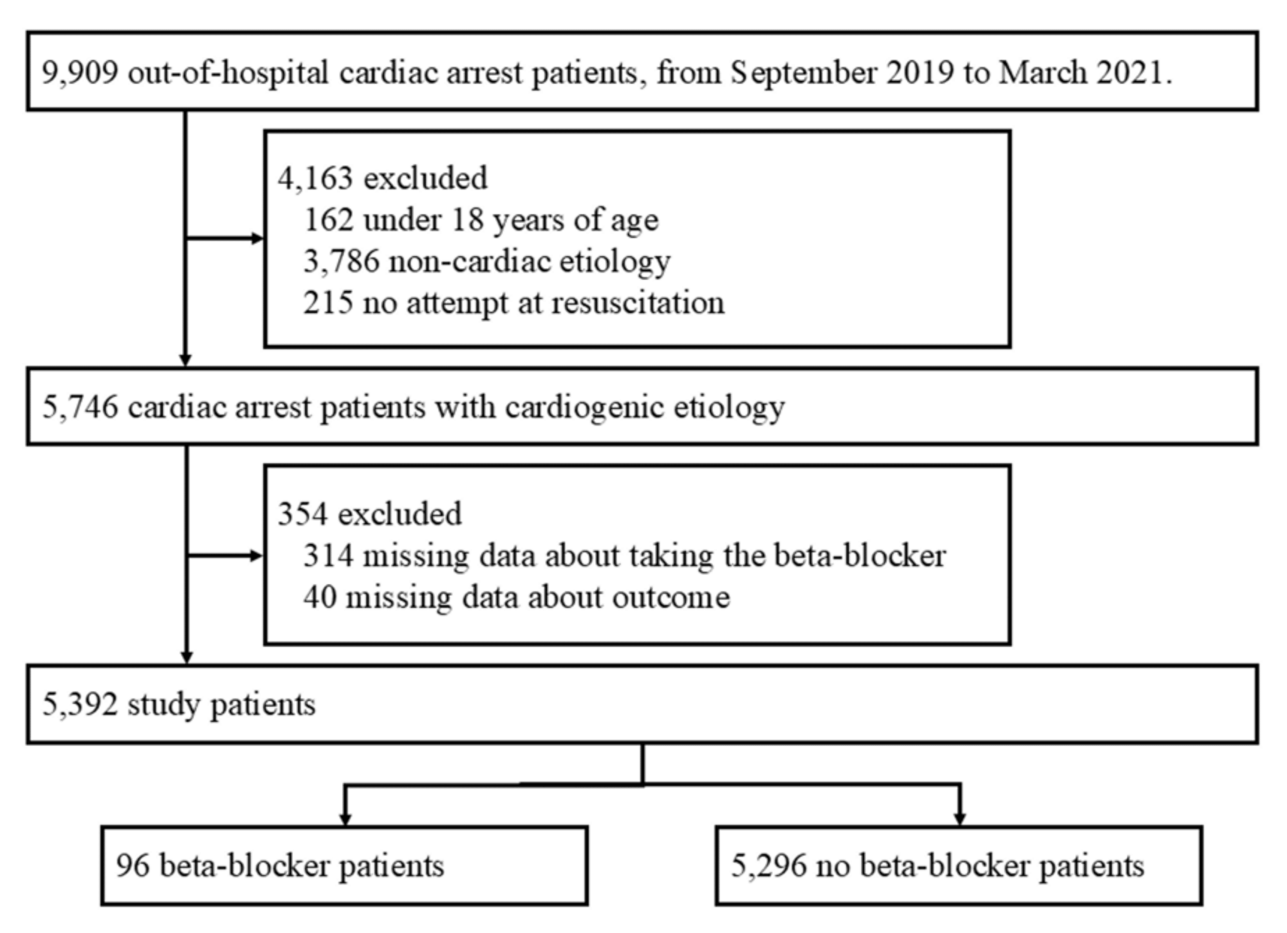
Flow diagram of the study population

A total of 9,909 cardiac arrest patients were enrolled in the SOS-KANTO 2017 study. Of these, 5,746 adult patients with cardiogenic cardiac arrest were extracted, and 5,392 patients were analyzed in our study after the exclusion process (Figure 1). Regarding the multiple imputations, the Clinical Frailty Scale had the largest proportion of missing data (24.9%), while the missing data rates for the other variables were all below 6%.

With regard to the baseline characteristics of the patients in this study, the mean age was 73.7 (SD, 14.7) years, 3,417 (63.4%) were men, 2,303 (44.9%) had a witnessed cardiac arrest, and 2,298 (45.3%) were treated with bystander CPR. Of the 5,392 patients, 96 (1.8%) took beta-blockers, and 5,296 (98.2%) did not take beta-blockers before OHCA (Table 1). Patients in the beta-blocker group more frequently had witnessed cardiac arrest (61.7% vs. 44.6%, respectively), an initial rhythm with VF (33.0% vs. 11.0%, respectively), and a higher prevalence of preexisting disease than those in the non-beta-blocker group at baseline (Table 1). The distribution of baseline covariates between the study groups was well balanced after overlap weighting for age, sex, witnessed arrest, bystander CPR, initial rhythm, etiology of cardiac arrest, the Clinical Frailty Scale, and each preexisting disease (Table 1).

**Table 1:** Baseline characteristics of the beta-blocker and non-beta-blocker groups SD: standard deviation; CPR: cardiopulmonary resuscitation; CPC: cerebral performance category

Characteristics	Unweighted			Overlap weighted		
	Beta-blocker (n = 96)	No beta-blocker (n = 5,296)	Standardized difference	Beta-blocker	No beta-blocker	Standardized difference
Age, years (SD)	73.1 (12.2)	73.7 (14.7)	0.048	73.1 (12.5)	73.1 (13.4)	<0.001
Sex, n (%) or % male	67 (69.8)	3,350 (63.3)	0.139	68.9	68.9	<0.001
Witnessed arrest, n (%) or %	58 (61.7)	2,245 (44.6)	0.347	63.1	63.1	<0.001
Bystander CPR, n (%) or %	48 (51.6)	2,250 (45.2)	0.128	50.0	50.0	<0.001
Initial rhythm, n (%) or %						
Ventricular fibrillation	31 (33.0)	539 (11.0)	0.849	29.2	27.3	<0.001
Pulseless ventricular tachycardia	1 (1.1)	20 (0.4)		0.6	2.5	
Pulseless electrical activity	27 (28.7)	1,063 (21.6)		30.0	28.6	
Asystole	23 (24.5)	3,004 (61.1)		26.1	30.8	
Others	12 (12.8)	289 (5.9)		14.1	10.7	
Etiology, n (%) or %						
Acute coronary syndrome	22 (22.9)	553 (10.6)	0.860	21.5	28.5	<0.001
Other heart disease	38 (39.6)	680 (13.0)		37.9	23.9	
Presumed cardiogenic	36 (37.5)	3,987 (84.7)		40.6	47.7	
Clinical Frailty Scale, n (%) or %						
Very fit	2 (2.4)	289 (7.4)	0.574	2.8	6.3	<0.001
Well	9 (11.0)	792 (20.4)		11.3	13.8	
Managing well	31 (37.8)	1,181 (30.4)		36.9	35.0	
Vulnerable	22 (26.8)	571 (14.7)		26.7	17.5	
Mildly frail	10 (12.2)	317 (8.2)		9.5	9.4	
Moderately frail	3 (3.7)	298 (7.7)		5.0	7.5	
Severely frail	4 (4.9)	300 (7.7)		6.1	7.9	
Very severely frail	0 (0.0)	100 (2.6)		0.5	2.0	
Terminally ill	1 (1.2)	34 (0.9)		1.2	0.7	
Medical history, n (%) or %						
Myocardial infarction	34 (36.6)	105 (2.0)	0.975	29.8	29.8	<0.001
Congestive heart failure	38 (42.2)	72 (1.4)	1.139	30.9	30.9	<0.001
Peripheral vascular disease	6 (6.5)	14 (0.3)	0.349	5.0	5.0	<0.001
Cerebrovascular disease	12 (12.6)	94 (1.8)	0.429	11.0	11.0	<0.001
Diabetes	30 (31.9)	170 (3.2)	0.814	31.7	31.7	<0.001
Renal disease	22 (23.4)	62 (1.2)	0.720	20.7	20.7	<0.001
Hypertension	59 (62.8)	281 (5.3)	1.525	55.0	55.0	<0.001
Atrial fibrillation	19 (20.4)	49 (0.9)	0.666	14.5	14.5	<0.001
Survival at 30 days, n (%) or %	34 (35.4)	351 (6.6)	0.755	33.4	31.9	-
CPC at 30 days, n (%) or %						
1	15 (16.1)	158 (3.0)	0.693	14.2	16.0	<0.001
2	8 (8.6)	53 (1.0)		9.8	6.6	
3	3 (3.2)	39 (0.7)		3.3	4.2	
4	3 (3.2)	60 (1.1)		3.9	4.8	
5	64 (68.8)	4,933 (94.1)		68.7	68.4	

The comparisons of survival and favorable neurological outcomes between the beta-blocker group and the non-beta-blocker group after overlap weighting are shown in Table 2. After overlap weighting, 33.4% of patients in the beta-blocker group and 31.9% of patients in the non-beta-blocker group ended up surviving. In the overlap weighting analysis, beta-blocker use was not associated with survival (odds ratio, OR, 1.07; 95% confidence interval, CI, 0.64-1.81) (Table 2). Beta-blocker use was also not associated with favorable neurological outcomes (OR, 1.09; 95% CI, 0.61-1.95) (Table 2). In the sensitivity analysis, propensity score matching results also showed no significant difference in survival (OR, 0.87; 95% CI, 0.48-1.58) and favorable neurological outcomes (OR, 0.73; 95% CI, 0.39-1.39) (Table 2).

**Table 2:** Clinical outcomes of survival and favorable neurological outcomes at 30 days between the beta-blocker and non-beta-blocker groups OR: odds ratio; CI: confidence interval

Outcomes	OR (95% CI)
Survival at 30 days	
After multiple imputation and overlap weighting	1.07 (0.64-1.81)
After multiple imputation and propensity score matching	0.87 (0.48-1.58)
Favorable neurological outcome at 30 days	
After multiple imputation and overlap weighting	1.09 (0.61-1.95)
After multiple imputation and propensity score matching	0.73 (0.39-1.39)

In the subgroup analysis, we extracted the data from patients with a history of cardiovascular disease. These patient characteristics are shown in Table 3. After overlap weighting, there was no significant difference in outcomes between the beta-blocker group and the non-beta-blocker group (Table 4).

**Table 3:** Baseline characteristics of the beta-blocker and non-beta-blocker groups in patients with cardiovascular disease SD: standard deviation; CPR: cardiopulmonary resuscitation; CPC: cerebral performance category

Characteristics	Unweighted			Overlap weighted		
	Beta-blocker (n = 85)	Non-beta-blocker (n = 403)	Standardized difference	Beta-blocker	Non-beta-blocker	Standardized difference
Age, years (SD)	72.6 (11.9)	71.6 (14.0)	0.084	72.4 (12.2)	72.4 (13.4)	<0.001
Sex, n (%) or % male	57 (67.1)	286 (71.0)	0.085	67.3	67.3	<0.001
Witnessed arrest, n (%) or %	53 (63.9)	273 (69.1)	0.112	66.6	66.6	<0.001
Bystander CPR, n (%) or %	44 (53.7)	207 (53.1)	0.012	52.9	52.9	<0.001
Initial rhythm, n (%) or %						
Ventricular fibrillation	29 (34.9)	114 (29.2)	0.178	32.2	31.6	<0.001
Pulseless ventricular tachycardia	1 (1.2)	4 (1.0)		1.2	1.9	
Pulseless electrical activity	23 (27.7)	100 (25.6)		29.0	28.7	
Asystole	19 (22.9)	101 (25.9)		22.8	24.0	
Others	11 (13.3)	71 (18.2)		14.7	13.8	
Etiology, n (%) or %						
Acute coronary syndrome	21 (24.7)	123 (30.8)	0.379	25.2	32.9	<0.001
Other heart disease	36 (42.4)	99 (24.8)		41.6	26.3	
Presumed cardiogenic	28 (32.9)	177 (44.4)		33.2	40.9	
Clinical Frailty Scale, n (%) or %						
Very fit	2 (2.7)	28 (8.2)	0.419	3.0	6.5	<0.001
Well	8 (11.0)	49 (14.3)		11.1	10.8	
Managing well	30 (41.1)	129 (37.7)		40.4	38.4	
Vulnerable	18 (24.7)	57 (16.7)		24.1	19.4	
Mildly frail	8 (11.0)	29 (8.5)		9.7	9.3	
Moderately frail	2 (2.7)	20 (5.8)		3.5	6.4	
Severely frail	4 (5.5)	25 (7.3)		6.9	7.5	
Very severely frail	0 (0.0)	4 (1.2)		0.1	1.3	
Terminally ill	1 (1.4)	1 (0.3)		1.3	0.5	
Medical history, n (%) or %						
Myocardial infarction	34 (41.0)	105 (26.2)	0.315	37.2	37.2	<0.001
Congestive heart failure	38 (46.9)	72 (18.3)	0.640	37.0	37.0	<0.001
Peripheral vascular disease	6 (7.2)	9 (2.3)	0.235	5.8	5.8	<0.001
Cerebrovascular disease	11 (12.9)	60 (15.1)	0.062	12.7	12.7	<0.001
Diabetes	28 (33.3)	112 (28.0)	0.116	34.7	34.7	<0.001
Renal disease	21 (25.0)	41 (10.2)	0.395	22.5	22.5	<0.001
Hypertension	59 (70.2)	281 (70.1)	0.004	68.7	68.7	<0.001
Atrial fibrillation	19 (22.9)	49 (12.3)	0.280	18.3	18.3	<0.001
Survival at 30 days, n (%) or %	32 (37.6)	148 (36.7)	0.019	38.0	37.1	-
CPC at 30 days, n (%) or %						
1	14 (17.1)	61 (16.0)	0.187	16.9	17.9	<0.001
2	7 (8.5)	22 (5.8)		9.7	7.1	
3	3 (3.7)	19 (5.0)		4.0	5.1	
4	3 (3.7)	26 (6.8)		4.8	6.5	
5	55 (67.1)	254 (66.5)		64.6	63.4	

**Table 4:** Clinical outcomes between the beta-blocker and non-beta-blocker groups in patients with cardiovascular disease OR: odds ratio; CI: confidence interval

Outcome	OR (95% CI)
Survival at 30 days	
After multiple imputation and overlap weighting	1.04 (0.61-1.75)
Favorable neurological outcome at 30 days	
After multiple imputation and overlap weighting	1.09 (0.61-1.93)

## Discussion

This study demonstrated that patients taking beta-blockers had similar survival rates and favorable neurological outcomes at 30 days to those not taking beta-blockers prior to OHCA. Furthermore, even in the subgroup analyses of patients with underlying heart disease, there were no significant differences in outcomes based on beta-blocker use.

Previous studies have reported that the use of beta-blockers may influence the distribution of shockable and nonshockable rhythms in OHCA patients. The presence of initial shockable rhythms is a predictive factor for favorable outcomes in OHCA patients, and increasing nonshockable rhythms could potentially worsen the prognosis. In a single-center cohort study of 478 patients, Youngquist et al. reported that beta-blocker use was associated with increasing nonshockable rhythms [6]. However, when Granfeldt et al. analyzed OHCA patients using a population-based registry in Denmark [20], they found that several cardiovascular drugs were significantly associated with shockable rhythms, but there was no significant association observed for beta-blockers [20]. Barcella et al. also examined the influence of different types of beta-blockers on the initial rhythm in OHCA patients based on registries from the Netherlands and Denmark [7]. They reported that nonselective beta-blockers, excluding beta-1 selective beta-blockers, were associated with nonshockable rhythms in OHCA patients [7]. It is important to note that these studies examined various factors influencing the initial rhythm but did not conduct any prognosis analysis [6,7,20].

Czarnecki et al. investigated the association between beta-blocker use and shockable rhythms and mortality in OHCA patients aged 65 and older using the Toronto OHCA registry [8]. Their research population had a higher mean age compared to our study. Yet, similar patient characteristics were observed with regard to witnessed cardiac arrest, the presence of bystander CPR, shockable initial rhythms, and mortality at 30 days [8]. Furthermore, beta-blocker use was not significantly associated with a decrease in shockable rhythms or mortality at 30 days in elderly OHCA patients [8], which was in line with our findings for adult OHCA patients.

Beta-blockers are widely prescribed since they can reduce the risk of hospitalization and mortality in patients with a history of myocardial infarction or heart failure [9-12]. In this study, only 96 patients (1.8%) were taking beta-blockers before OHCA. Previous research studies have reported approximately 20%-30% beta-blocker usage rates in OHCA patients [6-8]. Since the proportion of patients taking beta-blockers in our study was lower than in the literature, we considered using a different patient population. We extracted the data of patients with a history of heart disease for subgroup analysis. In the subgroup analysis, 85 of 488 patients (17.4%) with a history of cardiovascular disease, such as myocardial infarction, heart failure, hypertension, and atrial fibrillation, were on beta-blockers (Table 3). No significant differences in outcomes were found between the beta-blocker and non-beta-blocker groups in both the subgroup analysis and primary analysis (Table 4).

Our study used the OHCA registry in Japan, which has one of the highest aging rates in the world [21]. A rapidly aging population is an issue that many countries may potentially face in the future. With increasing age, the burden of cardiovascular disease is expected to rise [22,23], and the prescription of beta-blockers for conditions such as hypertension and heart failure is also expected to increase. Since beta-blockers are commonly used medications, it is essential to assess not only their effectiveness but also any adverse reactions. Notably, our study confirmed that beta-blockers did not worsen the prognosis of patients with OHCA.

Limitations

The limitation of this study is its causal inference due to the observational design. First, beta-blocker use was determined using patient medical records. However, it is possible that some patients in the non-beta-blocker group may have been misclassified due to incomplete or missing documentation of medication use. If medication records were unavailable or ambiguous, these cases were recorded as missing data and excluded from analysis. Nevertheless, there remains a potential limitation regarding the accuracy of the medication use classification. While incomplete patient information may have contributed to this disparity, our study prospectively collected data on beta-blocker use, making it more reliable than retrospective analyses. Second, we were unable to adjust for unmeasured covariates, although we did perform adjustments for patient characteristics using overlap weighting based on propensity scores. For example, we could not adjust for details of the beta-blocker prescriptions, such as time of drug initiation, specific types of medications, dosage information, and adherence to the prescribed medication, because those data were not available. Instead, we adjusted for the patient's underlying condition, which could have explained the beta-blocker usage. Third, we did not evaluate the treatment and care after hospitalization. There remains some controversy related to various types of postresuscitation treatment, and differences in intensive care management between facilities could have influenced the findings. Finally, this study evaluated survival rates and neurological outcomes in patients at 30 days; however, the long-term prognosis is unknown and warrants further investigation.

## Conclusions

Prescription of beta-blockers is expected to increase in super-aging societies. Thus, it is essential to confirm their safety with OHCA patients to ensure favorable clinical outcomes. Our study showed that beta-blocker use was not associated with survival and favorable neurological outcomes among OHCA patients in the SOS-KANTO 2017 study. Therefore, they can be safely prescribed to this patient population.
